# The Practical Management of Intractable Haematuria Within the National Health Service of the United Kingdom: A Literature Review

**DOI:** 10.7759/cureus.96083

**Published:** 2025-11-04

**Authors:** Toni Mihailidis, Kim Davenport

**Affiliations:** 1 Urology, Cheltenham General Hospital, Cheltenham, GBR

**Keywords:** haemorrhagic cystitis, intractable haematuria, national health service, radiation-induced cystitis, united kingdom

## Abstract

The aim of this article is to provide an updated review of the practical management of intractable haematuria in the National Health Service (NHS) of the United Kingdom (UK). A literature search was performed using the PubMed Advanced Search Builder. The terms ‘Intractable Haematuria’ OR ‘Haemorrhagic Cystitis’ OR ‘Radiation Cystitis’ were entered in a joint search as terms in a given publication’s title only. This generated 295 papers in total, which were manually screened for suitability. There were no specific inclusion or exclusion criteria. We liaised with senior pharmacists in our Medicine Information department to assess product availability. Based on the best available evidence, we propose a new treatment escalation plan for the management of intractable haematuria based on the safety profile and product availability in the UK. After initial management, first-line treatment could include intravesical hyaluronic acid, tranexamic acid, carboprost, adrenaline, or PuraStat®. Second-line treatment could include hyperbaric oxygen therapy or super selective embolisation. Percutaneous diversion and cystectomy represent last resorts. Not all proposed options for the management of intractable haematuria published in the literature are of practical use in the UK, and each has its own merits and disadvantages. The evidence base for the majority of treatment modalities recommended in the literature is overall weak, with a lack of appropriate randomised controlled trials to support their use. More research is required on available treatments and potentially novel treatments in order to drive a robust national census to serve the often morbid patient cohort presenting with intractable haematuria in the UK.

## Introduction and background

Intractable haematuria may be defined as haematuria which is persistent or recurrent, being refractory to initial management. It has several causes, including infection, malignancy, and previous radiotherapy [1]. It may be potentially life-threatening and having a clear understanding of the management options available is vital [2]. The aim of this article is to provide an updated review of the management of intractable haematuria in the National Health Service (NHS) of the United Kingdom (UK). Whilst there are many publications covering a breadth of management options for intractable haematuria, none have considered the practicality, availability, and applicability of these treatments specifically in the UK. 

A widely accepted approach to the treatment of intractable haematuria usually commences conservatively with three-way catheter insertion, thorough bladder washout and continuous bladder irrigation with saline [3]. Concurrent blood transfusion is administered according to patient's haemoglobin levels. If haematuria continues after 24 - 48 hours, then proceeding to rigid cystoscopy, bladder washout, and cystodiathermy or laser ablation is an appropriate next step. Upper tract causes must be excluded with imaging, such as a computed tomography urothelial phase. The majority of cases of intractable haematuria occur secondary to haemorrhagic cystitis, of which there are many causes, including malignancy, pelvic radiotherapy and/or chemotherapy (e.g. cyclophosphamide or ifosfamide), infection (such as E. coli), and autoimmune conditions (such as systemic lupus erythematosus, rheumatoid arthritis, granulomatosis with polyangiitis/Wegener’s granulomatosis or secondary to their treatment with drugs such as cyclophosphamide), amongst others [4,5]. It is a challenging and morbid condition, often resulting in prolonged hospitalisation and, albeit rarely, mortality, particularly amongst immunosuppressed patients [6].

Radiation cystitis is the most common cause of haemorrhagic cystitis, occurring in up to 10% of patients treated with radical radiotherapy of the pelvis, with a mean time of the onset of symptoms from treatment being approximately 35 months [7,8]. Whilst its pathophysiology is not completely understood, it is initially characterised by loss of the glycosaminoglycan layer and damaged vascular urothelium due to bladder wall oedema and inflammation. These changes lead to a progressive endarteritis over time, resulting in bladder ischaemia, necrosis, neovascularisation, tissue sloughing and hence haematuria. The end-stage is a fibrotic bladder of reduced capacity [7,9].

The main controversy and challenges of treatment surround subsequent management when conservative or initial surgical measures are unsuccessful, hence the absolute importance of this review. Accordingly, this article reviews first-, second-, and third-line management options for the management of intractable haematuria in the United Kingdom in the absence of a current nationally agreed but much needed guideline or consensus. We will explore the merits and disadvantages of each treatment modality in addition to their practicality and availability in the United Kingdom prior to suggesting a possible escalation ladder for each line of management based on best available evidence outlined in this article. This is essential for the management of a morbid condition presenting in high volume to urologists globally [1].

A literature search was performed using the PubMed Advanced Search Builder. The terms ‘Intractable Haematuria’ OR ‘Haemorrhagic Cystitis’ OR ‘Radiation Cystitis’ were entered in a joint search as terms in a given publication’s title. This generated 295 papers in total, which were manually screened for suitability. Though titles which were obviously irrelevant were discarded as they did not focus on the topic of intractable haematuria/haemorrhagic cystitis (>200 articles), there were no specific inclusion or exclusion criteria. Regarding the availability of different treatment options, we liaised with senior pharmacists in our Medicine Information department to subsequently assess product availability in the UK.

## Review

**Figure 1:**
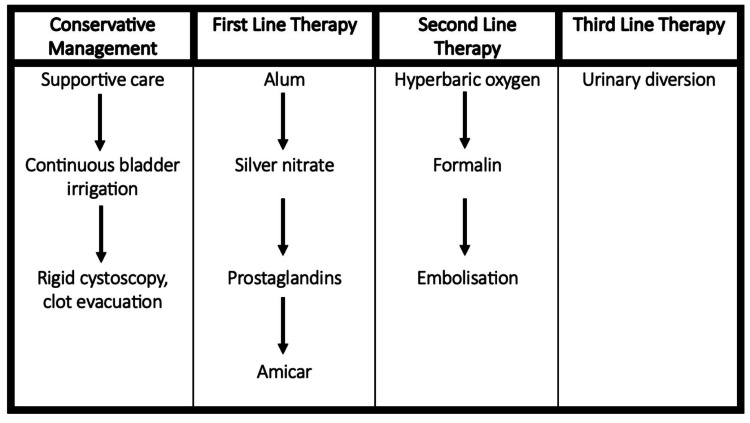
A figure to show a proposed treatment escalation plan according to D’Amico et al. This figure delineates a proposed model for the management of intractable haematuria. Whilst helpful, it is not a reflection of how intractable haematuria would be managed in the United Kingdom (UK). Reproduced from ref [2] under Creative Commons Attribution License 4.0 (CC-BY 4.0).

A wide variety of treatment options have been proposed, with the latest escalation ladder published by D’amico et al. (Figure 1) [2].

First-line management options

Alum

One treatment option widely cited in literature is the use of intravesical Alum [10]. This is potassium aluminium sulfate which was available in a 1% solution (50g in five litres of sterile water), to be instilled into the bladder through a three-way catheter over 48 - 72 hours. Its mechanism of action is thought to be via stimulation of vasoconstriction of bladder wall vasculature and sclerosis of capillary endothelium. Response rates of 60 - 100% are reported in the literature [11,12]. It has shown some success in treating both chemotherapy and radiation-induced haemorrhagic cystitis [13]. However, Alum was last available for therapeutic purposes in the NHS in 2019, and its use for medical purposes has been discontinued in the UK, though the reasons for this are unclear. There are safety concerns around the use of Alum, such as the risk of aluminium toxicity, particularly amongst patients with chronic kidney disease (CKD), which can lead to acute encephalopathy and osteomalacia [14,15]. Other side effects include fever, transient delirium, urinary tract infections, suprapubic pain and bladder spasm [5]. There have been no published randomised controlled trials (RCTs) or meta-analyses on its use for intractable haematuria. It does not feature in guidelines published by either the British Association of Urological Surgeons (BAUS) or the European Urology Association (EAU). Whilst it remains available to purchase commercially as a chemical, it is not listed in the British National Formulary (BNF) for any clinical indication. At present, it is therefore not a safe or practical option for the management of intractable haematuria in the UK.

Silver Nitrate

Silver nitrate causes chemical cauterisation through the binding of free silver ions to tissues, obstructing blood vessels secondary to eschar formation [16]. It is used to control bleeding in other clinical settings, particularly in the control of epistaxis [17]. Given its effects of chemical cauterisation, vesicoureteric reflux must be ruled out prior to commencing treatment through a cystogram [17]. One study of nine patients assessed the outcomes of intravesical silver nitrate for the treatment of intractable haematuria. In this study, the concentration of silver nitrate was gradually increased from 1:7500 (0.01%) to 1:1000 (0.1%) over the course of two to four days. Irrigation was discontinued if hematuria did not improve after reaching these maximum dosage thresholds. The study found that 100% patients continued to experience haematuria despite intravesical silver nitrate and required additional treatment needs, such as blood transfusions, concluding that it is an ineffective treatment for intractable haematuria [18]. Whilst this study reported no complications, more historical studies have reported secondary anuria, presumed to be caused by ureteric ulceration and oedema [19]. Whilst listed in the BNF, it is only listed as a topical treatment (as a stick or cream) for common warts, verrucas, and granulomas; it is not licensed for intravesical use to treat haematuria. Side effects are rare, but include argyria and methaemoglobinaemia [20]. Silver nitrate is widely available in UK practice, though clinicians must be wary that its intravesical use with irrigation to treat haematuria would be off-license and appropriate patient consent should be obtained. Even so, its limited evidence and poor results render it an unlikely choice.

Prostaglandins

Prostaglandins are available in a variety of forms, including PGE1 and PGE2, but the form clinically accessible in the UK is PGF2-alpha. The commercially available form of PGF2-alpha in the UK is known as carboprost. Currently, it is only licensed in the management of post-partum haemorrhage for patients unresponsive to ergometrine or oxytocin, and therefore any intravesical use is rendered off-license [21]. Furthermore, it is only licensed as an intramuscular injection and is available as a 250 micrograms per millilitre solution. This can in theory be diluted to constitute bladder instillations but there is no existing pre-constituted commercial medicinal product of this nature. The mechanism of action of prostaglandins specifically in treating haematuria is not well understood, though it is postulated that they in part induce vasoconstriction and smooth muscle contraction [22]. Several studies using PGF2-alpha/carboprost specifically to treat cyclophosphamide-induced haemorrhagic cystitis report up to a 100% success rate with no complications or adverse outcomes [22,23]. Several administration protocols have been described and they generally involve continuous bladder irrigation, such as 8 - 10 mg/L of carboprost run at a rate of 100 ml/hour for 10 hours [24]. Studies conducted using other forms of prostaglandins (PGE1 and PGE2) report similar beneficial effects [25,26]. These are all small studies with limited generalisability. Carboprost is only contraindicated in patients with cardiac or pulmonary disease [21]. Overall, carboprost seems a safe, practical, and available option for use in UK practice, however the evidence to support its use is weak and its clinical use would be off-license.

Amicar

Another treatment option recommended by D’ Amico et al. [2] is intravesical epsilon-aminocaproic acid (EACA), also known as Amicar [27]. It confers its action by binding plasminogen to fibrin, preventing the activation of plasmin, thereby reducing fibrinolysis [28,29]. Studies report a response rate of up to 92% [28]. As with Alum, there have been no published RCTs or meta-analyses, and it is not recommended by any specific UK or European guidelines. Amicar is not listed in the BNF and is not available in the UK. It was once available on the UK drug market, but only as an oral agent and it was not licensed for haematuria. Whilst it is not exactly clear why, one potential reason is that studies comparing EACA to tranexamic acid (TXA; another fibrinolytic agent) have illustrated that EACA leads to comparatively more blood loss during surgery [30]. Compared with EACA, TXA demonstrates 10-fold increased affinity for plasminogen, rendering TXA the drug of choice [31]. Accordingly, research has explored the use of intravesical TXA to treat intractable haematuria.

Alternative first-line management options in the UK

Intravesical Tranexamic Acid

TXA is a widely available drug in the UK and has shown success in treating gross haematuria in pilot RCTs [32]. TXA inhibits the activation of plasminogen to plasmin, ultimately preventing the formation of fibrin [33]. Hence, it is termed an anti-fibrinolytic agent. A double-blind RCT on 50 patients instilled 500mg of TXA dissolved in 100ml of normal saline via a clamped three-way catheter for 15 minutes. The results showed that intravesical TXA significantly reduced the amount of irrigation required and was effective at reducing haematuria. However, there was no reduction in transfusion requirements or haemoglobin level [32]. There were no adverse outcomes documented. Thus, whilst not incorporated into official guidelines, there have been randomised trials to support its use and TXA is widely available in the NHS.

Intravesical Adrenaline

Another option that may be more suitable for UK practice is intravesical adrenaline. One study injected 150ml of diluted adrenaline (1:10000) into the bladder cystoscopically, followed by bladder irrigation with 1:100000 diluted adrenaline for 24 hours [34]. The authors found a success rate of 86.7%, with patients not requiring any further additional treatment within one month (p<0.01). There were no adverse outcomes and patient observations remained unchanged during and after intravesical administration. Adrenaline is widely available in the UK and is not currently listed on a drug-shortage list [35], rendering it a potentially suitable option in the management of intractable haematuria in the UK. However, there is a paucity of research on this topic and more research is required in order to assess its safety profile with direct intravesical administration before it can be safely incorporated into official guidelines. 

Intravesical Hyaluronic Acid

iAluRil is a treatment option used specifically for radiation cystitis, which is a common cause of haematuria [36]. It is a combination of hyaluronic acid and chondroitin sulfate, both of which are normal components of the bladder mucosa. It helps to restore the glycosaminoglycan layer of the bladder and studies have shown its prophylactic efficacy in preventing against radiation cystitis [36,37]. iAluRil can therefore be used as a preventative measure when the haematuria has resolved, but there is no current evidence to support its use as a primary treatment. Hyaluronic acid in isolation, however, has demonstrated efficacy and been shown to be as effective as hyperbaric oxygen therapy in treating intractable haematuria in one RCT involving 36 patients with 18 months follow-up [38]. It was well tolerated in patients who had received it with the main side effect of urinary tract infection due to multiple catheterisations in 42.8% (P = 0.034) of patients. The current evidence base for hyperbaric oxygen therapy (HBOT) as treatment for intractable haematuria is overall stronger, however.

PuraStat®

PuraStat® is a new, emerging option in the treatment of intractable haematuria. PuraStat® is a synthetic, haemostatic hydrogel used to manage endoscopic bleeding in clinical settings, in particular gastrointestinal bleeding [39]. In one multi-centre pilot study involving 111 patients with gastrointestinal bleeding, haemostatic control was achieved in 94% of patients [39]. Indeed, PuraStat® has gone on to show efficacy in treating patients with severe, refractory radiation proctitis [40]. Applying this potential to the genitourinary tract, a single case report published this year on a 73-year-old male patient reported promising effects. Following failed initial treatment of a patient with intractable haematuria secondary to radiation cystitis with endoscopic washout and CystiStat®, PuraStat® was endoscopically introduced through a flexible cystoscope. 0.5ml was administered to each bleeding point seen, to a total of 3ml in this case [41]. This patient was subsequently scheduled for further application once every four weeks. There was only short-term data available on this treatment option, with no supporting medium to long-term outcome data available at present. A second case report in the United Kingdom showed similar findings: a 73-year-old male patient underwent a bladder neck incision in the private sector with multiple presentations with VH [42]. Previous interventions, including with iAluRil, were unsuccessful. Air cystoscopy was used to apply 7.5ml of PuraStat® to the bladder neck. Haematuria resolved and there was no ongoing haematuria at two-month follow up. The patient did however develop a recurrence of LUTS due to a bladder neck contracture diagnosed on flexible cystoscopy. PuraStat® is therefore a potentially viable option in the treatment of intractable haematuria in the United Kingdom but more studies are required in order to determine its safety profile and long-term outcomes.

Second-line management options

Hyperbaric Oxygen Therapy

HBOT is thought to work through stem cell mobilisation, reduction of inflammation, and neoangiogenesis [43]. It has been proposed as a treatment for intractable haematuria, particularly in cases of radiation-induced cystitis. A phase 2-3 RCT randomised 42 patients to receiving HBOT for radiation-induced cystitis [44]. This excluded patients whose ongoing haematuria required a blood transfusion 500ml in the preceding four weeks. In this regime, 100% oxygen was inhaled at a pressure of 240-250 kPa for 80 - 90 minutes a session for 30 - 40 sessions. The results showed an improvement in patient symptoms, as measured by the Expanded Prostate Index Composite (EPIC) score, particularly symptoms of 'urinary bother,' which includes irritative lower urinary tract symptoms and urinary incontinence [44]. Furthermore, in the aforementioned RCT, the effects of HBOT were compared with intravesical instillation of hyaluronic acid in treating visible haematuria in patient with haemorrhagic cystitis (n = 36) [38]. The RCT found that visible haematuria and urinary frequency improved in both groups at 18 months follow-up. More specifically in the HBOT arm, nine out of 20 patients experience complete resolution of macroscopic haematuria at 18 months follow-up. Whilst there are additional published prospective trials, there are no other published RCTs assessing the efficacy of HBOT in treating intractable haematuria.

There are a number of factors rendering HBOT of limited use in the UK at present. There are few centres offering HBOT for haematuria, treatment regimens are expensive, intensive and prolonged requiring patients to travel long distances regularly for a number of weeks. HBOT is not suitable for all patients and has important absolute contraindications. These include patients with bullous lung disease/large pulmonary cysts, patients with uncontrolled or poorly controlled chronic obstructive pulmonary disease, asthmatics, and previous thoracic surgery, and patients with pneumothoraxes without a chest drain, amongst others [45]. It has also been noted to have a number of side effects, including otalgia (up to 17%), otalgic barotrauma (up to 4% -13%), generalised seizures (up to 0.5%), vertigo, dizziness, anxiety (secondary to claustrophobia; up to 25%), nasal barotrauma (0.5%), and paraesthesia [44,46]. As evidenced in the literature, HBOT works over a prolonged period of time, with delayed benefits [44,47]. HBOT is very expensive, with prices as high as £200 per session, depending on the duration of treatment and the indication. Regarding this, caution must be taken with private, non-regulated sites where there may be insufficient scrutiny of patient suitability, a lack of properly qualified medical supervision, and where HBOT protocols may not be proven to be effective. It is not routinely commissioned in the NHS for radiation cystitis but is available, at time of writing, at Guy’s and St Thomas’ NHS Foundation Trust for local patients and for out-of-area referrals [48]. Treatment regimens at these centres vary. For example, the hyperbaric unit at Whipps Cross University Hospital, which accepts both private and NHS emergency and elective referrals, follows a protocol of 40 sessions, spread over five days a week [49]. It is also available in a small, select number of other trusts, which includes James Paget University Hospital Foundation Trust and the Torbay and South Devon NHS Foundation Trust, but intractable haematuria is currently not listed as an accepted indication and reason for referral [50,51]. Thus, HBOT may be a treatment option for intractable haematuria in the future, dependent on location and individual trust and/or local authority commissioning.

Formalin

Another cited option for the management of intractable haematuria is Formalin (Formaldehyde 37%) [52-54]. It is thought to act via bladder capillary occlusion, resulting in necrosis of layers of urothelium [52]. Typically, a 1% solution is instilled into the bladder via a urinary catheter for 15 minutes with light traction applied to prevent its exposure to the urethra. Studies report variable success rates, with the most recent cohort study reporting a success rate of 75%, defined by the number of patients who experienced complete resolution of haematuria [54]. However, like Alum, Formalin is not available in the UK, it is not listed in the BNF and it is not recommended by any formal guidelines for intractable haematuria. In practice, it is associated with significant risks. In the aforementioned most recent study, one patient underwent cystectomy due to bladder injury from intravesical Formalin instillation [54]. In more historical studies, urinary diversion was required in 40% of patients due to complications associated with Formalin intravesical treatment [55]. Additional risks cited throughout these studies include bladder dysfunction, vesicoureteric reflux, ureteric strictures, and renal failure - serious risks with life-long complications. These risks are dose-dependent, meaning that the risks increase as the concentration of Formalin administered increases [52]. Finally, formaldehyde is a recognised carcinogen, with many publications on this topic [56,57] and there is currently no published data with sufficient follow-up to show whether or not there is an increased risk of bladder carcinogenesis following intravesical administration for formaldehyde. Accordingly, formaldehyde is now classified as a chemical, not a drug in the UK, and therefore cannot be purchased in Pharmacy. Thus, whilst widely cited in literature, it is not a suitable option for the management of intractable haematuria in the UK.

Super Selective Arterial Embolisation

One treatment modality for intractable haematuria with a growing evidence base is super selective arterial embolisation of vesical branches of the internal iliac artery. This is offered in services with specialised Interventional Radiology (IR) availability and in patients who have failed less invasive therapies [58,59]. It can be performed under local anaesthetic, rendering it a suitable option for frail patients in whom a general anaesthetic would not be suitable. Whilst there are no published RCTs on the use of embolisation to treat intractable haematuria, one prospective study of nine patients with urological malignancy found a success rate of 100% with resolution of haematuria within 48 hours [60]. There have been many other studies on this topic, compiled in a recent systematic review [58]. Complications of embolisation include post embolisation syndrome, a syndrome comprising nausea, vomiting, gluteal pain, and tissue necrosis [59]. Ischaemic complications secondary to embolisation of the internal iliac artery are possible but can be reduced by the use of super selective embolisation [56]. Of all treatments discussed thus far, embolisation is the only treatment modality recommended by the National Institute for Health and Care Excellence (NICE) guidelines, specifically for intractable haematuria in the context of bladder cancer [61]. It is unclear exactly how many centres offer this at present. The main drawback in the future will likely be the availability and limited capacity of IR services in the UK.

Third-line management options

Percutaneous Urinary Diversion and Total Cystectomy

Percutaneous urinary diversion is a more invasive solution to intractable haematuria. The mechanism of action of urinary diversion in treating intractable haematuria is thought to be via the reduction of urokinase, reducing bleeding by reducing the activation of plasminogen to plasmin [62]. A single-centre retrospective observational study in 24 patients reported a 71% success rate, defined as a complete resolution of haematuria [62]. Complications reported in these papers were those related to nephrostomy insertion in general, including dislodgement and infection. A more invasive option yet would be a total cystectomy: this is a last resort option in extreme cases of intractable haematuria and up to 35% of patients who undergo nephrostomy insertion ultimately go on to cystectomy [63,64]. One study of 21 patients undergoing cystectomy for haematuria revealed that 42% of patients experienced severe complications, with a 19-day mortality of 16%. This was likely due to the comorbid nature and baseline clinical condition of a patient requiring such significant surgical intervention [63]. Thus, whilst available and a theoretically practical option, it is highly risky with a significant mortality rate. Both of these invasive options would, however, be possible as a last-resort treatment in the UK.

Recommendations for management of intractable haematuria in the UK

**Figure 2:**
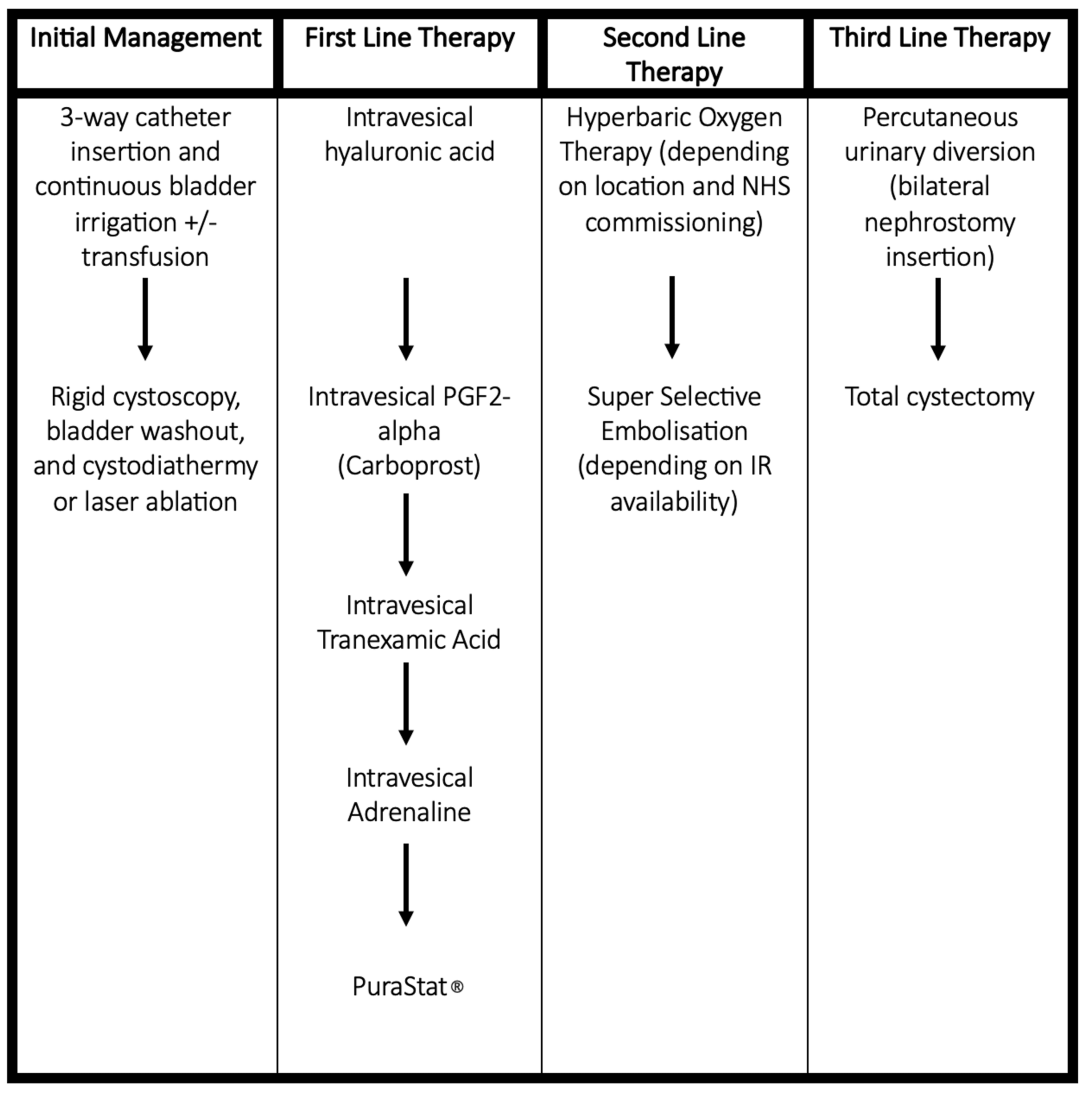
A figure to show the proposed treatment escalation plan for the management of intractable haematuria in the United Kingdom This figure is our proposed model for the management of intractable haematuria in the United Kingdom in the absence of any other consensus or national guideline based on the evidence available and product availability.

Based on the above, a practical treatment algorithm for the management of intractable haematuria in the NHS based on product safety and availability is shown in Figure 2.

## Conclusions

The management of intractable haematuria poses a challenge for urologists across the UK. Whilst many options have been proposed, not all are safe, practical and/or available in actual clinical practice in the NHS. This article has delineated potential practical options available for the management of intractable haematuria in the UK. The evidence base for the majority of these treatment modalities is overall weak and often historic, with a lack of appropriate RCTs to support their use. More research is required on available treatments and potentially novel treatments in order to drive a robust national census to serve the often morbid patient cohort presenting with intractable haematuria in the UK.
